# Systems-Level Modeling of Cancer-Fibroblast Interaction

**DOI:** 10.1371/journal.pone.0006888

**Published:** 2009-09-03

**Authors:** Raymond C. Wadlow, Ben S. Wittner, S. Aidan Finley, Henry Bergquist, Rabi Upadhyay, Stephen Finn, Massimo Loda, Umar Mahmood, Sridhar Ramaswamy

**Affiliations:** 1 Massachusetts General Hospital Cancer Center, Boston, Massachusetts, United States of America; 2 Department of Medicine, Harvard Medical School, Boston, Massachusetts, United States of America; 3 Broad Institute of Harvard and MIT, Cambridge, Massachusetts, United States of America; 4 Harvard Stem Cell Institute, Cambridge, Massachusetts, United States of America; 5 Department of Radiology, Center for Molecular Imaging Research, Massachusetts General Hospital, Boston, Massachusetts, United States of America; 6 Department of Medical Oncology, Center for Molecular Oncologic Pathology, Dana-Farber Cancer Institute, Brigham and Women's Hospital, Boston, Massachusetts, United States of America; University of Nottingham, United Kingdom

## Abstract

Cancer cells interact with surrounding stromal fibroblasts during tumorigenesis, but the complex molecular rules that govern these interactions remain poorly understood thus hindering the development of therapeutic strategies to target cancer stroma. We have taken a mathematical approach to begin defining these rules by performing the first large-scale quantitative analysis of fibroblast effects on cancer cell proliferation across more than four hundred heterotypic cell line pairings. Systems-level modeling of this complex dataset using singular value decomposition revealed that normal tissue fibroblasts variably express at least two functionally distinct activities, one which reflects transcriptional programs associated with activated mesenchymal cells, that act either coordinately or at cross-purposes to modulate cancer cell proliferation. These findings suggest that quantitative approaches may prove useful for identifying organizational principles that govern complex heterotypic cell-cell interactions in cancer and other contexts.

## Introduction

Cancer cells interact dynamically with surrounding stromal cells. Among the many relevant cell types within cancer stroma, fibroblasts appear to function prominently [1]. However, we lack a clear understanding of how molecular and cellular heterogeneity within this cell type functionally contributes to cancer initiation and progression [2]. In part, this is due to the experimental challenges inherent in studying multi-cellular interactions. While increasingly sophisticated animal models are being used to define discrete mechanisms by which fibroblasts contribute to tumor progression, these models are not well-suited for systematic discovery across multiple genetic and epigenetic contexts [3]–[6]. An alternative experimental approach involves analyzing the interaction of dissociated cancer cells and fibroblasts in vitro [7]–[11]. This approach has the potential to enable systematic and unbiased molecular screening for new stromal targets that can subsequently be validated in more physiologically relevant systems.

In vitro approaches to studying cellular interactions are generally limited by the choice of specific cells, culture conditions, and assays. The ideal system would examine functional interactions between different primary cancer cell and fibroblast populations co-derived from the same tumors. However, primary human cancer cells are notoriously difficult to propagate long-term ex vivo, and primary tumor-derived fibroblasts appear to undergo phenotypic changes in short-term culture [6]. In contrast, established cell lines are easily grown, relatively inexpensive, and readily available, thus representing a potentially useful and renewable resource for studying cancer-fibroblast interaction. In addition, culture conditions can influence cellular behavior but increasingly complex approaches that attempt to mimic physiologically relevant conditions, such as three-dimensional culture, scale poorly [12]. Finally, fibroblasts affect many aspects of cancer cell behavior including proliferation and survival, angiogenesis, invasion, metastasis, and drug resistance, but assays to score increasingly complex phenotypes can be challenging to implement in systematic studies.

We therefore performed a quantitative and integrated analysis using mathematical modeling of cancer cell proliferation in two-dimensional co-culture with a large number of normal fibroblast cell lines. These studies revealed that normal tissue fibroblasts variably express at least two functionally distinct activities in modulating cancer cell proliferation. Furthermore, transcriptional profiling of these different fibroblast populations revealed that at least one of these activities might relate to molecular programs that are present in activated mesenchyme. Systems-level modeling may thus be useful for identifying organizational principles that broadly underlie the interactions of cancer cells and fibroblasts, and may therefore inform systematic molecular studies of cancer-fibroblast interaction.

## Materials and Methods

### Cell lines and plasmid DNA

Cell lines were purchased from ATCC (Manassas, VA) or Coriell Cell Repositories (Camden, NJ). All fibroblast lines were used for co-cultures within 10 passages after purchase. Cancer and fibroblast cell lines were cultured in Dulbecco's Modified Eagle Medium (DMEM) with 10% fetal calf serum (FCS), L-glutamine (4 mM), penicillin (100 units/mL), and streptomycin (100 µg/mL). EGFP labeling of cancer cell lines was done using a third-generation lentiviral vector system. 293T cells were transfected using lipofectamine 2000 in a subconfluent 10-cm dish with the vector pCCLsin.PPT.hPGK (10 µg), into which EGFP had been cloned, as well as pMDLg/p packaging (7 µg) and VSV-G envelope encoding pMD.G (5 µg) plasmids. These plasmids were obtained from Rafaella Sordella at the MGH Center for Cancer Research and Luigi Naldini at the San Raffaele Telethon Institute for Gene Therapy. Viral supernatant was collected after 48 hours, filtered with a 0.45 micron syringe filter, and stored at −80°C. Cancer cell lines were infected in subconfluent wells of 24-well plates, using 300 µL of virus in 1 mL of DMEM culture media with 10% fetal calf serum. This protocol yielded infection rates in excess of 80% (determined by visual assessment using fluorescence microscopy). EGFP-negative cells were removed using a modified 5-laser Becton-Dickinson FACSDiVa with standard techniques as previously described [13].

### Quantitative co-cultures

2×10^4^ fibroblasts were seeded in 100 µL in at least 6 replicate wells in each of two 96 well plates and allowed to adhere into a confluent monolayer overnight. Subsequently 10^3^ EGFP-expressing cancer cells were seeded in an additional 50 µL into the fibroblast containing wells and into empty wells (150 µL total volume per well). A Spectramax M5 plate reader (Molecular Devices, Sunnyvale, CA) was used to obtain fluorescent readings approximately once daily for 14 days (excitation 477 nm, emission 515 nm). Thirty microliters of fresh media were added to each well on days 3, 6, 9, and 12. Wells containing fibroblasts or media alone, all with 150 µL of media per well on day 0, were measured in parallel and the values subtracted from co-culture and monoculture wells, respectively, to account for auto-fluorescence. All cultures were performed in DMEM with 10% FCS.

*Heterotypic xenografts:* Estradiol pellets (0.72 mg, 60-day release, Innovative Research of America, Sarasota, FL) were implanted into female nude mice (Charles River Laboratories) two days prior to xenograft injections. Mice were divided into 2 groups: 5 mice were injected with AG09877 fibroblasts and EGFP-expressing T47D breast cancer cells, and 5 mice were injected with AG04351 fibroblasts and EGFP-expressing T47D cells. Cells were trypsinized and re-suspended in Hank's Balanced Salt Solution at a concentration of 4×10^6^ million cells per 100 microliters. Animals anesthetized with isoflurane were injected with 4×10^6^ fibroblasts and 4×10^5^ cancer cells into the subcutaneous tissue over the mammary fat pad. EGFP signal was imaged and quantified using a bonSAI fluorescence optical imaging system immediately after injection, daily for four days, and then every 2–3 days. Mice were treated in compliance with MGH Institutional Animal Care and Use Committee regulations and sacrificed 43 days after injection. Tumor tissue was resected and flash frozen. Frozen sections were stained with hematoxylin and eosin or with anti-cytokeratin (CAM 5.2, Becton-Dickinson).

### Data processing

To quantify the effect of fibroblasts on the growth of cancer cells, we defined the mono-culture curve, 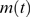, to be the difference on day *t* between the average fluorescence measurement for the wells with cancer cells but no fibroblasts and the average fluorescence measurement for the wells with media alone. We defined the co-culture curve, 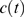, to be the difference on day *t* between the average fluorescence measurement for the wells with cancer cells and fibroblasts and the average fluorescence measurement for the wells with fibroblasts alone. We removed constant signal by subtracting the smaller day 0 value. Specifically, we let 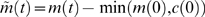 and let 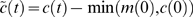. Co-culture ratios were defined as the ratio of the area under these two curves. Specifically, where *M* is the area under the 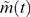 curve, we let 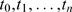 be the days for which we have measurements and interpolated linearly between measurement times. By the trapezoid rule,
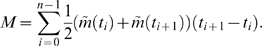



We defined *C* similarly to be the area under the 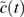 curve. We then defined the co-culture ratio, *E*, by
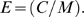



To compute confidence intervals (CIs) for *E*, we used the union of three bootstrap BC_a_ two-sided 95% CIs, each computed from 10,000 bootstrap samples [14]. The bootstrap samples are formed by first choosing with replacement from the two replicate 96-wells plates and by then choosing with replacement wells of each type (i.e., media-only, fibroblast, mono-culture, co-culture) from the chosen plates. The co-culture ratio was considered significant if the 95% CI for *E* was completely above or below one.

### Mathematical modeling

We hypothesized that a small number of functionally distinct interaction types underlie the large number of data points in the matrix of co-culture ratios. We suspected that if we could determine an optimal number, *N*, of simpler matrices with which to approximate the co-culture ratio matrix, then that optimal *N* would give us some idea of the number of functionally distinct interaction types at work and the matrices that comprise the approximation might give us some insight into the nature of those interactions.

A variety of methods exist to decompose a mathematical matrix into a sum of simpler matrices that are in some sense orthogonal or independent of one another [15]. Models based on singular value decomposition (SVD) or principal component analysis (PCA) have been most widely used across a broad range of biological, chemical, and physical sciences [16]. Examples include the deconvolution of anatomical or pathophysiological information from dynamic contrast-enhanced MRI and analysis of three-dimensional quantitative structure-activity relationships to predict the activity of candidate drugs [17]–[19]. We chose SVD for our analysis over PCA since PCA first centers the data by subtracting row or column means. However, the zero value of our matrix was set to equal an AUC ratio of 1, signifying the absence of an effect of co-culture on cancer cell proliferation. Thus to shift the zero value by subtracting means would have sacrificed its intrinsic meaning.

Decomposition methods are often coupled with cross-validation strategies to distinguish meaningful components from statistical noise [20]. The cross-validation strategy we chose to use employs the EM algorithm for the estimation of missing data [21] as detailed below.

Let *R* be the matrix of differences between the co-culture ratio matrix and 1, such that positive values of *R* correspond to co-cultures that stimulated cancer cell proliferation and negative values of *R* correspond to co-cultures that inhibited cancer cell proliferation. We wish to determine an optimal *N* for *R*. To do so, we use models the complexity of which increase with *N* to predict the value of each element of *R* from all the other elements of *R* and deem as optimal the *N* for which those predictions are maximally accurate.

Specifically, for any matrix *S*, let 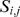 denote the element of *S* in the *i* ^th^ row and *j* ^th^ column, let 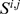 denote *S* with the element of *S* in the *i* ^th^ row and *j* ^th^ column missing and let 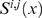 be 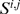 with the missing element filled in by *x*. Let 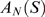 be the approximation of *S* gotten by adding the best *N* matrices of the singular value decomposition of *S* (i.e., those corresponding to the *N* largest singular values). In the case of missing data we define 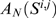 by the EM algorithm for the estimation of missing data as follows. Let
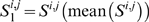
and let
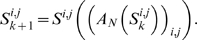



In our experience, this EM algorithm has always converged as *k* increases so we can let
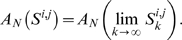



Let
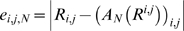
and let 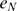 be the median of the 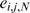 over all the *i* and *j*. The *N* for which 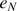 is minimal is deemed to be the optimal *N* for *R*.

### Gene expression profiling

Confluent plates of fibroblasts (replicating the conditions of co-culture) or cancer cells in log-phase growth were trypsinized, centrifuged into pellets, and flash frozen in liquid nitrogen. RNA was isolated with Qiagen RNeasy kits and profiled using Affymetrix HG-U133 Plus 2.0 microarrays with standard protocols [22].

## Results and Discussion

**Figure 1:**
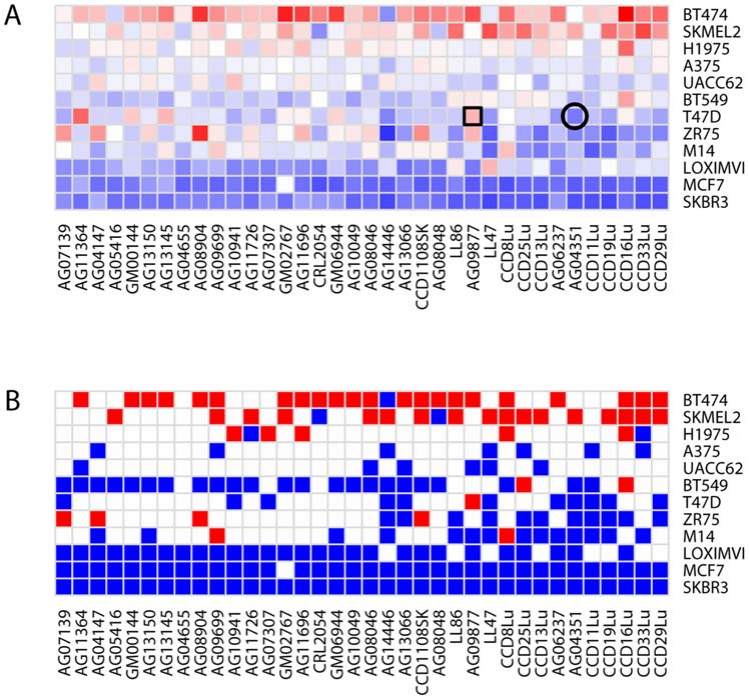
Quantitative analysis of cancer-fibroblast cell line interactions. A) Heat map representation of experimentally determined co-culture ratios for 432 cancer-fibroblast cell line interactions. Red refers to growth-stimulatory interactions and blue to growth-suppressive interactions. B) Interactions resulting in statistically significant growth stimulation of cancer cells (i.e. the lower bound of the 95% CI for the co-culture ratio is >1) are shown in red, and interactions resulting in statistically significant growth inhibition of cancer cells (i.e. the upper bound of the 95% CI for the co-culture ratio is <1) are shown in blue. The circle indicates the interaction between T47D and AG04351, and the square indicates the interaction between T47D and AG09877.

We first systematically co-cultured twelve human breast, melanoma, and lung cancer cell lines with thirty-six untransformed, human fibroblast cell lines derived from normal skin and lung (see Table S1 and Table S2 for details). Each cancer cell line was tagged with EGFP using lentiviral transduction to enable the composite quantification of cancer cell proliferation and survival over fourteen days using a simple plate reader-based assay system. For each cell line pairing (n = 432), which we examined in multiple replicates over independent experiments, we computed the ratio of the area under the EGFP curve for cancer cells grown in co-culture divided by the area under the curve for cancer cells grown alone (Figure 1A). We found that fifty-three of 432 cell line pairings (12%) were growth-stimulatory in absolute terms, defined by a 95% confidence interval with a lower bound greater than 1 (Figure 1B). In contrast, 176 (41%) were growth-inhibitory and 203 (47%) were null. These data demonstrate that only a minority of heterotypic cell line pairings yield increased cancer cell growth.

**Figure 2:**
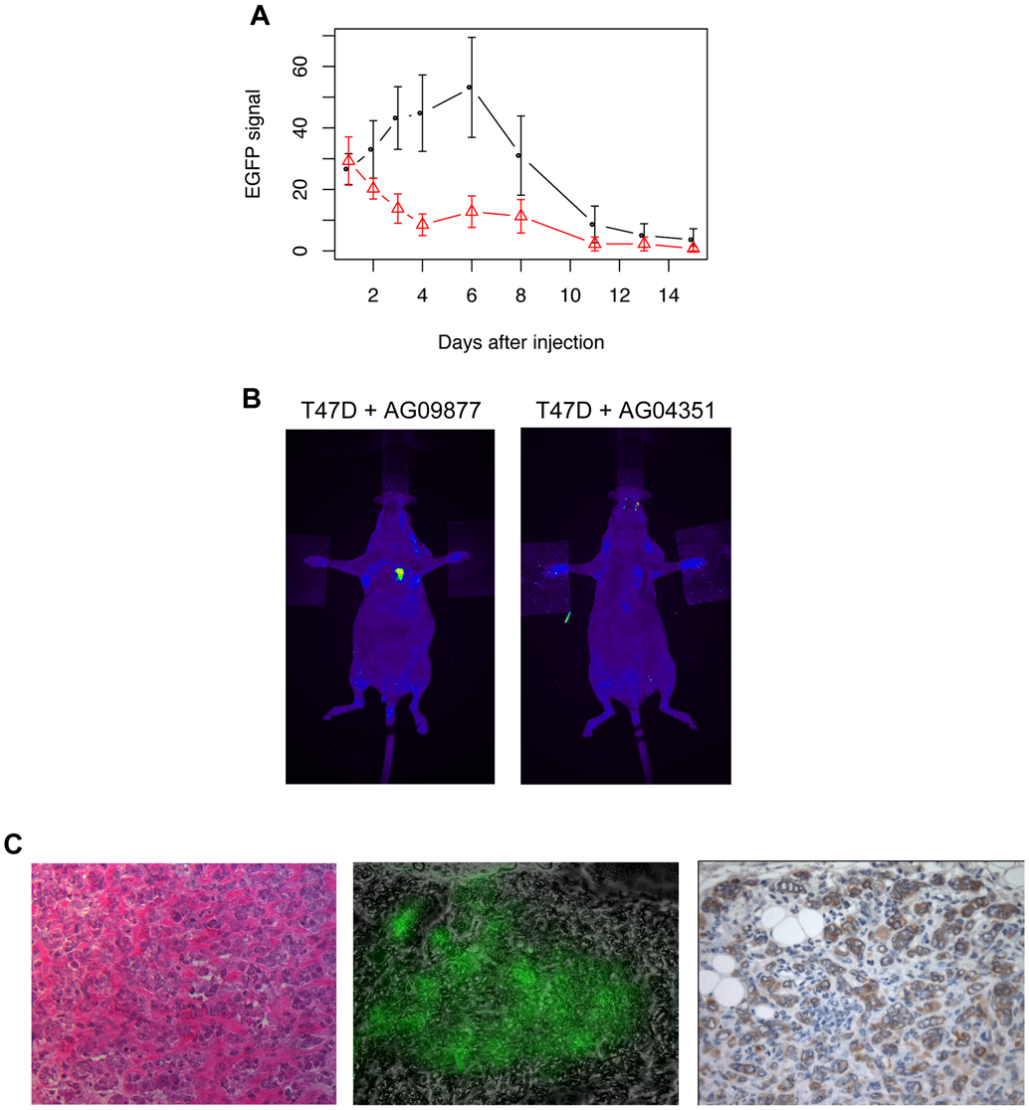
Cancer cell-fibroblast xenografts. A) EGFP signal from injections of EGFP-expressing T47D cells with AG09877 fibroblasts (5 mice, blue curve) or AG04351 fibroblasts (4 mice, red curve). Error bars represent standard error of the mean. B) Representative pictures of mice xenografted with each admixture taken 3 days after injection, with white arrows pointing to injection sites. C) Photomicrographs of a T47D-AG09877 tumor. From left to right: hematoxylin and eosin staining, GFP fluorescence, and immunohistochemistry for cytokeratin.

To explore the relevance of these co-cultures in vivo, we next focused on two specific cancer-fibroblast pairings that displayed opposing proliferative effects in vitro. Specifically, AG09877 stimulated T47D proliferation, while AG04351 was growth-inhibitory for this same cancer cell line. Co-injection of T47D cells and AG09877 fibroblasts subcutaneously in nude mice led to the formation of small tumors over one week (Figure 2A and 2B). In contrast, xenografting of these cancer cells with AG04351 fibroblasts did not result in tumor formation. These results confirmed that opposing effects of different fibroblast populations on cancer cell growth in vitro could also be observed in vivo. T47D alone is weakly tumorigenic (data not shown) [23], [24], and the fact that most induced tumors permanently regressed after the first week suggested that the growth-stimulatory effect of AG09877 fibroblasts was generally insufficient to sustain the prolonged growth of this cell line in vivo. Notably, however, one animal actually developed a persistent tumor over the course of six weeks. Pathologic examination of this single tumor revealed EGFP-positive cancer cells embedded within a significant desmoplastic stromal component (Figure 2C). While only a single experiment, this provocative result suggested that growth-stimulatory fibroblasts identified in vitro might be capable of exerting both transient and more sustained tumorigenic effects on adjacent cancer cells in vivo.

**Figure 3:**
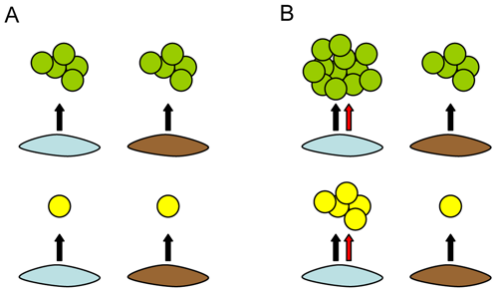
Cancer cell proliferation in co-culture is determined by properties of both cancer cells and fibroblasts. A) The growth response of cancer cell lines (circles) to fibroblasts (oblong shapes) is determined predominantly by the preprogrammed ability of cancer cells to proliferate in response to generic fibroblast signals (black arrows) shared in common across all fibroblast lines. Some cancer cell lines (green) are growth stimulated, while others (yellow) are unresponsive or growth-inhibited. B) Additional signals produced by subsets of fibroblasts (red arrows) add complexity by either further stimulating cancer cell proliferation (upper left panel) or compensating for the lack of response to generic signals (lower left panel). Although all signals in this schematic are defined as growth-stimulatory, they may also be growth-inhibitory thus adding further complexity.

We next aimed to identify organizational principles underlying the matrix of co-cultures that might provide insight into the biological determinants of cancer-fibroblast interaction. Systematically recreating the cellular interaction matrix using heterotypic xenografts might have offered further insight into the physiological relevance of individual pairings, but was not feasible for 432 different interactions. We therefore used a systems-level approach to characterize and our model in vitro data. To begin with, cursory inspection of the data in figure 1 revealed that cancer cell lines could be grouped into those that were predominantly inhibited (n = 3), largely inhibited (n = 6), or strongly stimulated (n = 3) by fibroblasts, suggesting that the growth response of a cancer cell line in a given stromal co-culture was pre-programmed and independent of the paired fibroblast line (e.g. Figure 3A). However, only SKBR3 displayed uniform responses across all fibroblast lines, implicating multiple fibroblast-specific contributions to cancer cell proliferation. In several cases this fibroblast contribution was sufficient to override the general predisposition of the cancer cell line, leading to a growth stimulatory interaction with an otherwise growth-inhibited cancer cell line or vice-versa (e.g. Figure 3B). Thus the growth response of cancer cell lines to stromal co-culture appeared to result from the combination of a dominant cancer cell-determined contribution and a smaller but often critically important fibroblast effect.

**Figure 4:**
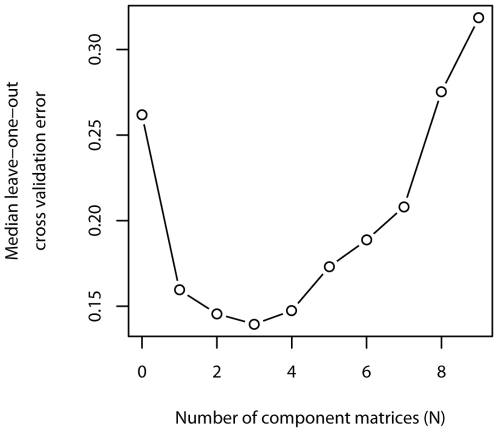
Cross-validation model. Median leave-one-out cross-validation error when the matrix of co-culture ratios is approximated by the sum of N component matrices.

**Figure 5:**
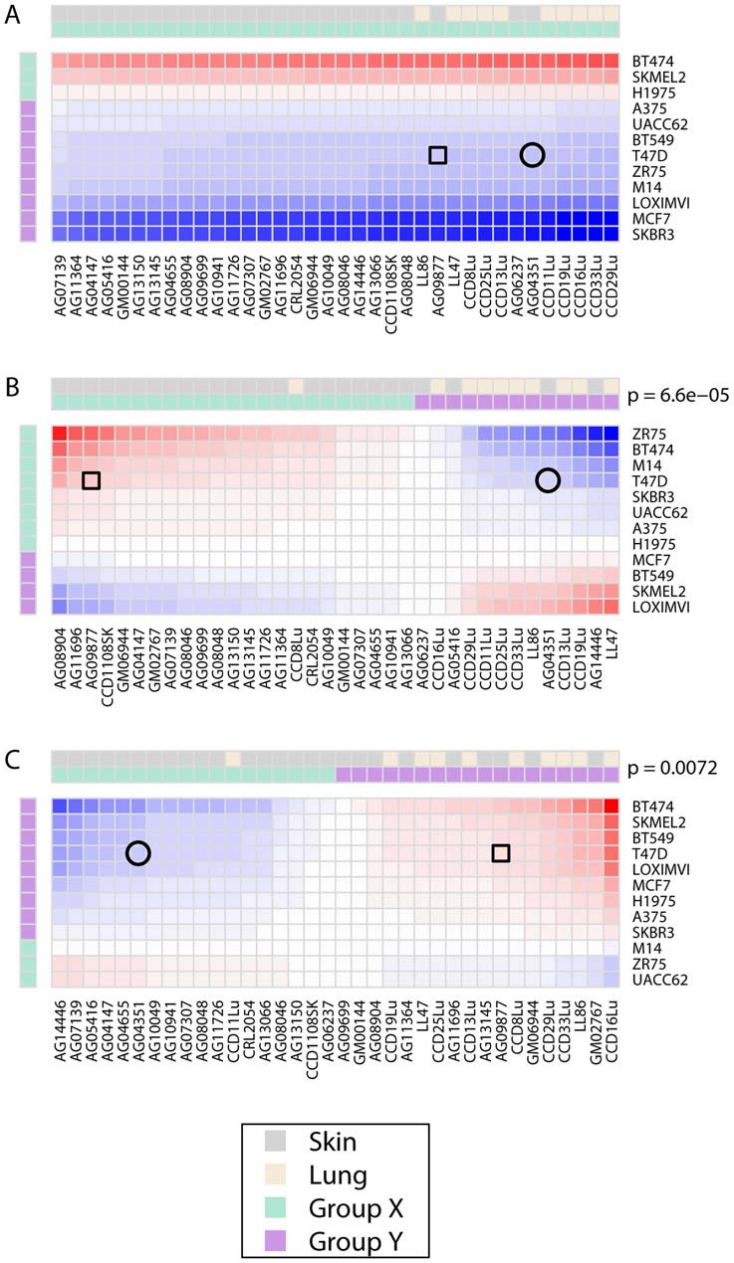
Mathematical modeling of cancer-fibroblast interaction. A)–C) Decomposition of the co-culture matrix into 3 component matrices. When applicable, cancer and fibroblast cell lines are divided into two groups (X, green, and Y, purple) such that interactions within the same group make growth-stimulatory (i.e. positive) contributions to the estimated co-culture ratio and interactions between opposite groups make growth-inhibitory (i.e. negative) contributions to the estimated co-culture ratio. P values refer to tissue of origin segregation between X and Y groups. Circles indicate the interaction between T47D and AG04351, and squares indicate the interaction between T47D and AG09877. Red refers to growth-stimulatory interactions and blue to growth-suppressive interactions, with intensity corresponding to the strength of the effect and scaled independently within each matrix.

Although Figure 3B schematically represents a parsimonious two signal model, the total number of interaction types could not be readily inferred through qualitative inspection of our dataset. We therefore used mathematical modeling based on singular value decomposition to ask whether the complex pattern of growth stimulation and inhibition we observed across this dataset resulted from a small, finite number of interaction types between cancer cells and fibroblasts. Decomposing the matrix of co-culture ratios into a sum of *N* component matrices, we used a leave-one-out cross-validation strategy to define the optimal value for *N* (Figure 4; see materials and methods for full details of the model). We found that the median cross-validation error reached its nadir with *N* = 3, suggesting that the net co-culture ratio for each cell line pairing resulted from the sum of interaction values represented by three distinct component matrices. Matrix A, which accounted for the majority of error reduction in the model, reflected the varying responsiveness of different cancer cell lines to generic stromal signals produced by fibroblasts (Figure 5A). For example, 9 of 12 cancer cell lines generally responded to fibroblasts with slowed growth, as exemplified by SKBR3 and MCF7, while three cancer cell lines were typically growth-stimulated, as evidenced by BT-474 and SK-MEL-2.

In contrast, matrix B and matrix C reflected distinct fibroblast activities that correlated significantly but imperfectly with a given fibroblast's tissue of origin (p = 6×10^−5^ and p = 0.0072 for matrices B and C respectively) (Figures 5B and 5C). In the space of each matrix, the fibroblasts were subdivided into two groups that interacted with distinct subsets of cancer cell lines to promote cancer cell proliferation (green vs. purple in figure 5). While the majority of cancer cell lines interacted cooperatively with the “skin-like” fibroblasts in matrix B, a different majority favored the “lung-like” fibroblasts in matrix C. Consequently, we were able to identify multiple examples in which the same fibroblast-cancer pairing resulted in positive effects on cancer cell proliferation in one matrix and negative effects in the other (e.g. T47D-AG07139), suggesting that fibroblasts could interact with cancer cells in at least two functionally distinct ways.

Despite the fact that the majority of error reduction in the model was contributed by matrix A, in some instances the effect size in matrices B and C in combination was sufficient to override that in matrix A. In fact, closer analysis revealed that the net effects of different fibroblasts on cancer cell proliferation could only be accurately determined by considering the *quantitative* contributions of effects from all three matrices. This is illustrated by interactions between the breast cancer cell line T47D and the two skin fibroblast cell lines AG09877 and AG04351 (denoted in Figure 1A and Figure 5A–C by squares and circles, respectively). Matrix A (Figure 5A) revealed that T47D was generally predisposed to growth suppression by all fibroblast cell lines. One plausible biological explanation for this could be the expression of a cell surface receptor for some cytostatic factor secreted by all fibroblasts. However, matrix B (Figure 5B) indicated that most skin fibroblast cell lines had a growth stimulatory activity for T47D, with a few notable exceptions including AG04351. In theory, this activity could be due to the expression by most skin fibroblasts of a specific mitogenic growth factor. In contrast, matrix C (Figure 5C) indicated that most skin fibroblast cell lines also had a second distinct growth inhibitory activity for T47D, with the important exception of AG09877 and several others. This activity could plausibly be attributed to the secretion by most skin fibroblasts of a specific growth inhibitory cytokine. Thus AG09877, by expressing the growth-stimulatory activity and lacking the growth inhibitory activity, made two functionally distinct growth-stimulatory contributions to T47D growth that were sufficient in combination to override the general predisposition of T47D to fibroblast-mediated growth suppression. In contrast, AG04351 only made growth suppressive contributions with respect to both fibroblast activities.

To gain insight into the molecular identity of these fibroblast activities, we isolated RNA from 36 fibroblast cell line monocultures and performed microarray-based gene expression profiling using Affymetrix gene chips. We first identified genes that were differentially expressed between skin and lung fibroblasts, between groups X and Y in matrix B, and between groups X and Y in matrix C. We then used gene set enrichment analysis (GSEA) [25] to identify gene sets enriched within each comparison. Using a false discovery rate (FDR) threshold of 0.25, we identified nine gene sets enriched in the skin vs. lung fibroblast distinction, including two that characterize the epithelial-to-mesenchymal transition (EMT) phenotype (Table 1) [26]. Although associated with higher FDRs, both sets were also enriched in the B_x_ (skin-like) vs. B_y_ (lung-like) distinction. In contrast, no gene sets were flagged in the C_x_ (skin-like) vs. C_y_ (lung-like) distinction, suggesting that this fibroblast activity may either reflect transcriptional differences only induced within the context of co-culture or non-transcriptional differences that could not be easily detected using microarray-based transcriptional profiling.

**Table 1:** Gene set enrichment analysis of skin vs. lung fibroblasts.

Gene set name	Brief description (Pubmed ID)	FDR q-value for enrichment in skin fibroblasts	FDR q-value rank for enrichment in skin fibroblasts	FDR q-value for enrichment in Group X of matrix B	FDR q-value rank for enrichment in Group X of matrix B	FDR q-value for enrichment in Group X of matrix C	FDR q-value rank for enrichment in Group X of matrix C
HTERT_DN	Downregulated in hTERT-immortalized fibroblasts vs. non-immortalized controls (12702554)	0.07	1	0.88	520	1.00	510
EMT_UP	Up-regulated during the TGFbeta-induced epithelial-to-mesenchymal transition (EMT) of Ras-transformed mouse mammary epithelial (EpH4) cells (EMT is representative of late-stage tumor progression and metastasis) (14562044)	0.15	2	0.30	1	0.91	430
JECHLINGER_EMT_UP	Genes upregulated for epithelial plasticity in tumor progression (14562044)	0.16	3	0.31	2	0.86	377
CIS_XPC_DN	Reduced expression in XPC-defective fibroblasts, compared to normal fibroblasts, following treatment with cisplatin (15107491)	0.18	4	0.77	28	0.86	374
CMV_24HRS_DN	Downregulated at 24 hrs following infection of primary human foreskin fibroblasts with CMV (9826724)	0.19	5	0.39	6	0.99	476
TSADAC_RKOEXP_UP	Genes with some basal expression and partially-methylated promoters, upregulated by the combination of TSA and DAC in RKO cells (11992124)	0.20	6	0.85	476	0.79	94
IDX_TSA_DN_CLUSTER5	Strongly down-regulated at 2–96 hours during differentiation of 3T3-L1 fibroblasts into adipocytes with IDX (insulin, dexamethasone and isobutylxanthine), vs. fibroblasts treated with IDX + TSA to prevent differentiation (cluster 5) (15033539)	0.21	7	0.32	3	1.00	510
FALT_BCLL_DN	Genes downregulated in VH3-21+ B-CLL (15817677)	0.21	8	0.81	91	0.87	399
CROMER_HYPOPHARYNGEAL_MET_VS_NON_UP	Genes increased in metastatic hypopharyngeal cancer tumours (14676830)	0.23	9	0.80	64	0.82	214

Nine gene sets enriched in skin vs. lung fibroblasts with corresponding false discovery rate (FDRs) q values and ranks. Also included are the FDR q values and ranks for each of these gene sets in the group X vs. Y distinction from matrices B and C of Figure 5.

EMT describes a coordinated program of cellular phenotypes increasingly recognized as crucial to the metastasis of carcinoma cells. These phenotypes include loss of epithelial cell polarity, increased cellular migration, and invasion into surrounding tissues [27]. Moreover, recent evidence indicates that EMT programs also regulate mesenchymal cell functions including angiogenesis [28]. Furthermore, the transcription factor Snail, a master regulator of EMT, is expressed in activated fibroblasts within healing wounds and at the tumor-stromal interface [29]. Our data thus suggested that EMT programs are preferentially expressed by many skin fibroblasts, perhaps serving as the molecular basis for one of the fibroblast activities (Type B) described by our quantitative model.

**Figure 6:**
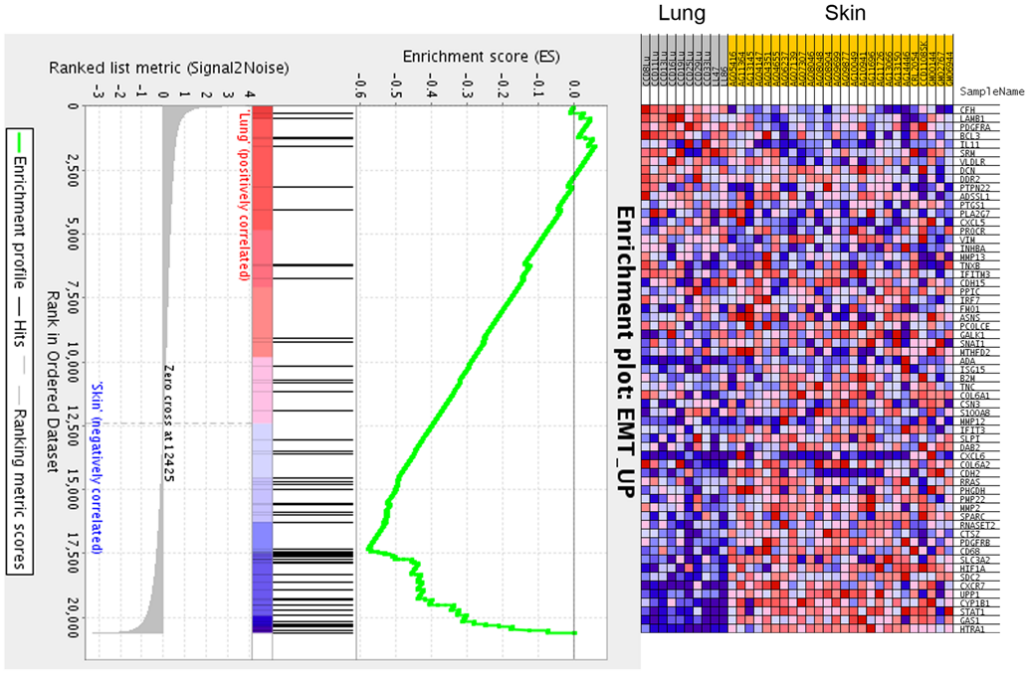
Enrichment of EMT genes in skin vs. lung fibroblasts. Heat map (right) and enrichment plot (left) for the EMT gene set from GSEA analysis of skin vs. lung fibroblasts [26].

Closer inspection of the two EMT gene sets reveals that many of the genes driving the enrichment in skin fibroblasts (i.e. the core enriched genes) are cell surface and secreted molecules that have been implicated in stromal contributions to tumor progression (Figure 6). For example, matrix metalloproteinases and cathepsins including MMP-2, MMP-12, and cathepsin Z are up-regulated in tumor stroma and promote cancer cell proliferation, migration, and invasion by degrading basement membranes and exposing cryptic migratory and growth signals [30]. Tenascin C is a matricellular protein that stimulates cancer cell proliferation and angiogenesis [31]. N-cadherin (CDH2) is expressed in the filopodia of myofibroblasts that migrate toward malignant cancer cells in a transforming growth factor beta-dependent manner [32]. SPARC (secreted protein acidic and rich in cysteine) is another stromal matrix protein that increases cancer cell invasion and that has been inversely correlated with survival in patients with pancreatic cancer [33], [34]. Stromal PDGFRB regulates interstitial fluid pressures and drug uptake within tumors [35]. Thus the same genes that regulate EMT in epithelial cancer cells also regulate functional contributions to malignant progression from the tumor stroma. Enriched expression of these genes in skin fibroblasts suggests tissue-specific preprogramming of mesenchymal populations for tumor stromal functionality.

Fibroblasts therefore appeared to display at least two distinct effects on the proliferative response of cancer cells in co-culture. Importantly, a *quantitative balance* between these two fibroblast activities and the general responsiveness of cancer cells to fibroblast signals largely determined the co-culture ratio for a particular cell line pairing. These independent fibroblast effects can apparently co-exist within individual fibroblast populations, functioning either cooperatively or at cross-purposes with respect to cancer cell growth. Intriguingly, our analysis suggests that both activities segregate fibroblasts largely according to tissue of origin. Moreover, microarray profiling indicated that one of these activities might reflect differential expression of a coordinated transcriptional program associated with activated mesenchymal cells. Further work will be required to fully characterize the molecular basis of each fibroblast activity and to evaluate the relevance of our findings to cancer-fibroblast interaction in real tumors.

This work was limited by the nature of the cell populations examined, insofar as established cancer cell lines and normal tissue fibroblasts may not completely phenocopy those cell populations that exist within an evolving human tumor. Additional studies with larger or more varied fibroblast panels might also identify new and different patterns of activity. Furthermore, these experiments included only 12 cancer cell lines. Experiments with a larger and more diverse cancer cell line panel may eventually reveal additional correlations between cancer cell tissue of origin or molecular subtype and growth response in co-culture. Finally, our SVD-based model was predicated on specific assumptions, including the supposition that serial deconvolutions of a matrix each account for maximum residual variability. This assumption may not always be accurate [36], and other algorithmic approaches may ultimately prove superior for modeling cell-cell interaction. However, this study offers a proof of principle that systems-level modeling may be useful to begin defining the organizational principles that govern cell-cell interaction. We anticipate that similar approaches applied to other cell types may be useful for studying heterotypic cell interaction both in cancer and other contexts.

Gene expression data have been deposited in NCBI's Gene Expression Omnibus [37] and are accessible through GEO Series accession number GSE17032.

## Supporting Information

Table S1 Cancer Cell Lines
(0.04 MB DOC)

Table S2 Fibroblast Cell Lines
(0.07 MB DOC)
