# Supplementary material for: Systems-Level Modeling of Cancer-Fibroblast Interaction
Source: PLoS One. 2009 Sep 3;4(9):e6888. doi: 10.1371/journal.pone.0006888 (PMC2731225; doi:10.1371/journal.pone.0006888)
Supplement: Table S1 — Cancer Cell Lines (0.04 MB DOC) [file pone.0006888.s001.doc]

Table S1

| **Cell Line** | **Source** | **Cell Type** | **Tissue** |
| --- | --- | --- | --- |
| MCF7 | ATCC | breast adeno | pleural effusion |
| SK-BR-3 | ATCC | breast adeno | pleural effusion |
| ZR-75-1 | ATCC | breast IDC | ascites fluid |
| BT-474 | ATCC | breast IDC | breast |
| BT-549 | ATCC | breast IDC | breast |
| T-47D | ATCC | breast IDC | pleural effusion |
| A375 | ATCC | melanoma | skin |
| UACC62 | NCI | melanoma | skin |
| UCLA-SO-M14 | NCI | melanoma | skin |
| LOX-IMVI | NCI | melanoma | skin |
| SK-MEL-2 | ATCC | melanoma | skin metastasis |
| H1975 | ATCC | lung adeno | lung |
