# Supplementary material for: Systems-Level Modeling of Cancer-Fibroblast Interaction
Source: PLoS One. 2009 Sep 3;4(9):e6888. doi: 10.1371/journal.pone.0006888 (PMC2731225; doi:10.1371/journal.pone.0006888)
Supplement: Table S2 — Fibroblast Cell Lines (0.07 MB DOC) [file pone.0006888.s002.doc]

Table S2

| **Cell Line** | **Source** | **Tissue** |
| --- | --- | --- |
| LL 47 | ATCC | lung |
| LL 86 | ATCC | lung |
| CCD-8Lu | ATCC | lung |
| CCD-11Lu | ATCC | lung |
| CCD-13Lu | ATCC | lung |
| CCD-16Lu | ATCC | lung |
| CCD-19Lu | ATCC | lung |
| CCD-25Lu | ATCC | lung |
| CCD-29Lu | ATCC | lung |
| CCD-33Lu | ATCC | lung |
| CCD-1058Sk | ATCC | skin |
| CCD-1108Sk | ATCC | skin |
| AG07139 | Coriell | skin |
| AG11364 | Coriell | skin |
| AG04147 | Coriell | skin |
| AG05416 | Coriell | skin |
| GM00144 | Coriell | skin |
| AG13150 | Coriell | skin |
| AG04655 | Coriell | skin |
| AG08904 | Coriell | skin |
| AG09699 | Coriell | skin |
| AG10941 | Coriell | skin |
| AG11726 | Coriell | skin |
| AG07307 | Coriell | skin |
| GM02767 | Coriell | skin |
| AG11696 | Coriell | skin |
| GM06944 | Coriell | skin |
| AG10049 | Coriell | skin |
| AG08046 | Coriell | skin |
| AG14446 | Coriell | skin |
| AG13066 | Coriell | skin |
| AG08048 | Coriell | skin |
| AG09877 | Coriell | skin |
| AG04351 | Coriell | skin |
| AG13145 | Coriell | skin |
| AG06237 | Coriell | skin |
